# The landscape of neuroscience research in Africa: current state, progress, and challenges; a perspective

**DOI:** 10.1097/MS9.0000000000001219

**Published:** 2023-09-01

**Authors:** Nicholas Aderinto, Muili Abdulbasit, Gbolahan Olatunji, Mariam Edun

**Affiliations:** a Department of Medicine and Surgery, Ladoke Akintola University of Technology; b Department of Medicine and Surgery, University of Ilorin, Ilorin, Nigeria

**Keywords:** Africa, current state, neuroscience research, progress

## Abstract

The field of neuroscience research in Africa has witnessed significant advancements in recent years, contributing to understanding the brain and neurological disorders. This paper provides an overview of the current state of neuroscience research in Africa, highlighting the progress made, ongoing efforts, and the challenges researchers face. Despite limited resources and funding constraints, African scientists have made remarkable strides in various areas of neuroscience. Collaborative networks and international partnerships have been crucial in advancing education, research infrastructure, and capacity building in the field. Institutions in countries such as Egypt, Ghana, South Africa, Nigeria, Tunisia, and Morocco have emerged as key hubs for neuroscience research, fostering a growing community of researchers dedicated to unravelling the complexities of the brain. Efforts in neuroscience research have encompassed diverse domains, including neurogenomics, neuroimaging, neurophysiology, neurorehabilitation, and neuroepidemiology. Studies have focused on investigating genetic factors underlying neurological conditions, exploring the neural mechanisms of cognition and behaviour, and developing innovative therapeutic approaches for neurological disorders. However, challenges persist in the African neuroscience research landscape. Limited funding remains a significant barrier, hindering the establishment of well-equipped laboratories, access to advanced technologies, and support for research projects. Addressing these concerns is crucial to ensure research outcomes’ integrity, validity, and relevance. Looking ahead, strategic interventions are required to address these challenges and further advance neuroscience research in Africa.

## Introduction

Highlights

- The anatomy of quadratus lumborum block is systematically introduced.
- The advantages and disadvantages of four methods of quadratus lumborum block are summarized.
- The application of quadratus lumborum block in different types of surgery is reported.

Advancing neuroscience research and education in Africa with far-reaching healthcare, education, and socioeconomic development implications is critical. With its diverse population, unique challenges, and untapped potential, Africa presents a promising landscape for exploring opportunities in neuroscience, addressing inherent challenges, and evaluating the impact of these advancements. The importance and relevance of advancing neuroscience in Africa must be considered. The African continent bears a heavy burden of neurological disorders, including epilepsy, stroke, neurodevelopmental disorders, and infectious diseases affecting the nervous system^1^. These conditions have profound implications for the health and well-being of African populations, leading to significant disability and socioeconomic burdens. However, the understanding of these disorders in the African context is limited, and context-specific research is essential to address the unique challenges faced by the region^2^.

Furthermore, the advancement of neuroscience research in Africa holds promise for improving healthcare outcomes. Researchers can develop tailored diagnostic tools, treatment strategies, and preventive measures by unravelling the intricacies of the nervous system and its involvement in disease processes. In a region where resources are often scarce, a focused and informed approach to neuroscience research can lead to cost-effective interventions that better serve the needs of African populations^3^. In addition to its impact on healthcare, investing in neuroscience education in Africa has broader implications for socioeconomic development. By nurturing a well-trained workforce of neuroscientists, clinicians, researchers, and educators, African countries can drive scientific progress, technological innovation, and the establishment of robust healthcare infrastructures. This, in turn, can attract domestic and international investments, foster collaborations with global institutions, and pave the way for sustainable development in the region. Despite these compelling reasons, a considerable gap exists in African neuroscience research and education. Limited resources, including funding, laboratory facilities, and advanced technologies, hinder the progress of scientific investigations. Moreover, the brain drain phenomenon, where skilled researchers and scientists migrate to other regions, exacerbates the need for more expertise within the continent. The need for comprehensive neuroscience curricula and training programs further impedes the development of a skilled workforce in Africa.

In light of these challenges, exploring the opportunities for advancing neuroscience in Africa is imperative. The current state of knowledge in neuroscience research in Africa remains limited, with a notable gap in understanding the challenges and opportunities faced by researchers in the region. While some studies have explored specific aspects of neuroscience in Africa, a comprehensive review synthesising the existing literature and highlighting the key gaps needs to be included. Understanding these gaps is crucial for identifying areas that require further investigation, addressing barriers to progress, and maximising the potential of neuroscience research in Africa. This paper examines the current landscape comprehensively, underscoring the importance of continued investment, collaboration, and support for advancing African neuroscience research and education.

## The current state of neuroscience research in Africa

Neuroscience research in Africa has shown a persistent upward trend, building upon a rich history that traces back to ancient Egypt. Although not on par with the Western world by all measures, neuroscience in Africa has been gaining momentum. In recent years, there has been a notable increase in awareness and literature on stroke, epilepsy, and other neurological disorders^4^. This surge in research can be attributed to collaborative efforts with researchers from developed countries, funding from local and international sources, and the growing accessibility of advanced imaging techniques such as functional magnetic resonance imaging^4^. Additionally, establishing organisations like the Society of Neuroscientists of Africa (SONA) and the International Brain Research Organization (IBRO) has substantially advanced neuroscience research across the continent.

The prevalence of neurological disorders in Africa is increasing, contributing significantly to the global disease burden. A 2016 study revealed that the absolute number of daily adjusted life years (DALYs) related to neurological disorders increased by 15% from 240 379 in 1990 to 276 143 in 2016^5^. Africa substantially contributes to these figures, with an absolute number of DALYs reaching 24 000 and an age-standardized DALY rate of 30 per 100 000 people. The observed 28% increase in DALYs highlights the continent’s growing burden of neurological disorders. Consequently, there is a pressing need to enhance the quality and quantity of neurological research conducted in Africa.

An analysis of neuroscience publications in Africa revealed that Egypt, South Africa, Nigeria, Morocco, and Tunisia have had the highest number of neuroscience publications since 1996^4^. The main focus areas in African neuroscience studies include neurodegeneration and damage, methods, excitability, synapses and glia, development, physiology, and behaviour. The visibility of African neuroscience publications varies, with some papers outperforming non-African publications regarding citation counts. However, citation rates differ across the continent, with articles from Southern Africa receiving more citations than those from West Africa. Moreover, only a few publications from African laboratories reach the top-tier journals with high-impact factors. While African neuroscience primarily relies on type 1 techniques, genetically tractable model systems still need to be improved. Nevertheless, diverse research on endemic medicinal plants across the continent presents opportunities for drug discovery and the exploration of natural medicinal products^6^.

In recent years, many African research institutions have been dedicated to studying neurological disorders, crucial in advancing neuroscience research on the continent. These institutions are at the forefront of scientific discovery and have significantly contributed to understanding and addressing African neurological diseases. The Neuroscience Institute at the University of Cape Town (UCT) in South Africa is one such institution. It was established to enhance neuroscience research and expand the country’s treatment options for neurological illnesses. The institute collaborates with the UCT and the Western Cape Government Department of Health to develop the Neuroscience Centre, which serves as a hub for academics and researchers in neuroscience. Equipped with cutting-edge technology, the centre brings together patient treatment, advocacy, teaching, and research under one roof. This collaborative approach fosters research advancements, enhances patient care, and promotes collaboration to tackle the burden of neurological diseases in South Africa and beyond.

The Institute for Advanced Medical Research and Training (IAMRAT) at the University of Ibadan is another notable institution. IAMRAT conducts research that addresses the health needs of the African continent and the people of Nigeria. The institute is dedicated to providing leadership in basic and applied medical research, coordinating research activities, facilitating the exchange of ideas, organising training courses and workshops, and attracting research grants from local and international funding agencies. IAMRAT’s Neurodegenerative Unit focuses on researching the causes and treatment of neurodegenerative diseases, including Alzheimer’s disease and Parkinson’s disease.

Furthermore, another prominent African research facility is the Extramural Unit (EMU) on the Genomics of Brain Disorders (GBD) at Stellenbosch University. The EMU adopts a systems biology approach to identify genomic biomarkers for various brain disorders. The EMU aims to contribute to genomics, neural signatures, and behavioural sciences through innovative methods such as multi-‘omics’ analysis, cognitive-affective assessments, and brain imaging techniques. By understanding the underlying causes of brain disorders, the unit strives to facilitate the development of new treatments. Additionally, the EMU focuses on securing funding, establishing collaborations, producing scholarly outputs, and engaging in community partnerships to further its research goals. These institutions represent just a few examples of the numerous facilities scattered across the continent, each making unique contributions to advancing neuroscience research in Africa. These efforts are crucial for improving our understanding of neurological disorders, developing effective treatments, and addressing African populations’ specific challenges. These institutions are pivotal in driving neuroscience research in Africa by fostering collaboration, securing funding, and leveraging innovative approaches.

It is important to note that the neuroscience research landscape in Africa is experiencing notable diversification, encompassing a wide range of focus areas and innovative approaches. Researchers across the continent are actively exploring various domains within neuroscience to enhance our understanding and improve the quality of life for individuals affected by neurological conditions. One prominent area of focus is neuroepidemiology, where studies aim to understand the distribution, determinants, and impact of neurological diseases within African populations. Researchers are delving into the prevalence, risk factors, and burden of conditions such as stroke, multiple sclerosis, and transverse myelitis. For instance, Prof. Owolabi Mayowa from the University of Ibadan has contributed significantly to this field, developing the ‘Seed of Life Model,’ a conceptual framework that encapsulates the holistic essence and quality of life^7^. Additionally, Prof. Mayowa’s work has led to the development of health-related quality of life in stroke patients, a globally available and multiculturally-validated quality-of-life measure designed for stroke patients^7^. These innovative measures provide valuable insights into the impact of stroke on patients’ lives and serve as a guide for interventions and treatment strategies. Another important area of research in Africa is the study of neuroimmunological diseases, including multiple sclerosis and transverse myelitis. Researchers such as Dr Hesham Abboud contribute to unravelling the mechanisms, risk factors, and optimal management strategies for these conditions^8^. Their work sheds light on the unique aspects of neuroimmunology in Africa, fostering better understanding and tailored approaches to diagnosis and treatment.

These examples highlight the current effort towards neuroscience research in Africa. The growing diversity of neuroscience research in Africa signifies a promising trajectory for addressing African populations’ unique challenges and needs. It underscores the importance of continued support and investment in neuroscience research endeavours on the continent.

## Opportunities for neuroscience advancement in Africa

Africa has vast potential for advancing neuroscience research on the continent and globally. As the birthplace of various hominin lineages, including modern humans, Africa is important in human evolution^9^. This rich genetic diversity offers a unique opportunity to delve deeper into the role of genetic factors in neurological conditions that are not yet fully understood. It also provides a platform to enhance the existing knowledge regarding certain neurological conditions, the pharmacodynamics of drugs, and precision medicine. The continent’s diverse habitats, flora, and fauna further contribute to the potential for comparative research. Africa’s wide variety of animal models presents exceptional opportunities to investigate brain health, neurological disorders transmission, and drug discovery. For instance, the African green monkey (Chlorocebus sabaeus) is an important animal model for studying neurodegenerative diseases^10^. The unique genetic and physiological similarities between the African green monkey and humans make it an ideal model to explore disease progression, evaluate therapeutic interventions, and develop novel treatments. Such research can yield invaluable insights into the understanding and treatment of nervous system diseases.

Compared to developed continents, Africa experiences a higher prevalence of traumatic brain injuries, spinal cord damage, and brain infections^11^. Researchers can identify neurobiomarkers and develop safe and effective treatments by studying these disorders and their common progression processes. This focused research has the potential to address the specific healthcare challenges faced by African populations and contribute to advancements in neuroscience. A notable study by researchers focused on understanding the genetic underpinnings of various cognitive abilities in African populations^12^. They explored the relationship between genes and different aspects of the brain, including memory, language, thinking, and spatial skills. The study identified specific gene variations, such as rs73485231 on chromosome 13, associated with enhanced memory, and rs140578927 on chromosome 6, linked to language skills. Additionally, they discovered the importance of the gene BIN1 in executive function tasks. However, the study found no strongly associated genes related to spatial skills. While providing valuable insights, further research is necessary to unravel the complex relationship between genes and cognitive abilities. Conducting more studies of this nature will benefit Africans and the global neuroscience community, advancing our understanding of the intricate interplay between genes and cognitive functions.

The presence of inadequate financing, infrastructure, and limited access to cutting-edge technologies in Africa has compelled African scientists to adopt creative approaches in neuroscience research. African researchers have demonstrated remarkable ingenuity, leading to the development of innovative techniques, interdisciplinary collaborations, and the exploration of low-cost diagnostic and therapeutic approaches. These endeavours have contributed to significant advancements in various branches of neuroscience study, including neuroimaging, electrophysiology, and genetic analysis.

Interdisciplinary partnerships have emerged as a driving force in African neuroscience research, fostering a comprehensive and holistic approach. By bringing together diverse perspectives and expertise, these collaborations enhance understanding of complex neurological phenomena. Moreover, African scientists have pioneered the creation of low-cost research tools and methodologies, often adapting existing methods or devising novel solutions tailored to their specific needs. This adaptability is exemplified in neuroimaging, electrophysiology, and genetic analysis, where African researchers have successfully developed affordable approaches to collect valuable data and contribute to the broader understanding of neuroscience. Noteworthy examples of African innovation in neuroscience include research on Xysmalobium undulatum extract, which has demonstrated the ability to inhibit A42 synthesis and promote non-amyloidogenic protein processing^13^. This finding holds potential implications for Alzheimer’s disease treatment. Additionally, African researchers have developed culturally relevant materials to improve the diagnosis of mental health conditions, ensuring that assessments and interventions align with the sociocultural context. In a pilot study conducted in Gambia, functional near-infrared spectroscopy was utilised to investigate brain function in cognitive disorders, showcasing the application of affordable methodologies in studying brain dynamics^14^.

These innovative approaches in neuroscience research in Africa not only address financial constraints but also provide valuable insights into neurological conditions. The affordability and adaptability of these methodologies enable researchers to gather data and expand the understanding of neuroscience in resource-limited settings. Importantly, the advancements made in African neuroscience have the potential to benefit not only the African population but also other regions facing similar challenges. The creative solutions and pioneering techniques developed in Africa contribute to the global scientific community and potentially shape future research endeavours in neuroscience worldwide.

## Challenges and limitations

Neuroscience research in Africa holds immense potential for understanding the complexities of the human brain, addressing neurological disorders, and advancing global knowledge in the field. However, it is crucial to acknowledge and address the challenges and limitations that impede the progress of neuroscience research on the continent.

Insufficient funding poses a formidable obstacle to neuroscience research in Africa, impeding the establishment of well-equipped laboratories, impinging upon access to advanced technologies, and restraining adequate support for research endeavours. This paucity of financial resources constrains the scale and breadth of research activities, thereby inhibiting the potential for transformative discoveries and impeding the advancement of scientific inquiry. The limited funding availability jeopardizes the capacity to establish state-of-the-art laboratories equipped with cutting-edge equipment and technologies essential for conducting sophisticated experiments and analyses. Deprived of these indispensable resources, researchers across Africa encounter impediments in unravelling the intricacies of the brain’s mechanisms and exploring innovative approaches to comprehending neurological disorders. For example, researchers studying the genetic basis of epilepsy have faced difficulties in securing funding for genome sequencing and genetic analysis. This financial constraint limits their ability to explore the underlying mechanisms of the disorder and identify potential therapeutic targets. Moreover, more financial support is needed to attract and retain talented researchers, who may be enticed by superior funding opportunities and career prospects elsewhere.

Consequently, the brain drain phenomenon exacerbates the depletion of scientific expertise within Africa, further hindering the progression of neuroscience research on the continent. Due to limited funding opportunities and inadequate support for research projects, highly skilled neuroscientists have sought career prospects and research positions abroad. As a result, collaborative efforts among local researchers have been hindered, as talented individuals contribute their expertise to foreign institutions. This brain drain depletes scientific expertise within Africa and diminishes the collaborative environment necessary for propelling neuroscience research forward.

The repercussions of insufficient funding extend beyond curtailing individual research projects, further impeding collaborative potential. Collaboration is indispensable in advancing neuroscience research, as it facilitates knowledge exchange, fosters multidisciplinary approaches, and enables the pooling of resources and expertise. However, the capacity to cultivate effective collaborations becomes compromised without sufficient financial support^15^. Researchers cannot dedicate time, resources, and travel expenses to foster meaningful collaborations. As a result, the collective advancement of neuroscience in Africa is diminished, as opportunities for knowledge exchange and interdisciplinary collaboration are hindered.

Similarly, the need for more essential resources and infrastructure undermines the capacity of African researchers to delve into the intricate workings of the brain and comprehensively investigate neurological disorders. Inadequate laboratory facilities and a dearth of cutting-edge technology limit the exploration of innovative approaches and methodologies, hindering the advancement of neuroscience research. Moreover, limited access to specialised equipment and advanced imaging techniques significantly restricts the ability to study the brain’s structure, function, and connectivity with precision and accuracy. These indispensable tools are pivotal for unravelling the complexities of neurological conditions and elucidating the underlying mechanisms at play. Such infrastructure is necessary to maintain the depth and breadth of investigations into neurological disorders, impeding the development of effective diagnostic and therapeutic strategies. Furthermore, Africa needs more research infrastructure to improve collaboration and knowledge exchange among researchers and institutions. Collaborative efforts are essential for fostering interdisciplinary approaches, pooling resources and expertise, and accelerating scientific progress. However, the absence of robust infrastructure hampers the establishment of collaborative networks and diminishes the collective potential for advancing neuroscience research on the continent.

The departure of skilled researchers to more developed countries exacerbates the existing limitations of research capacity in Africa. It creates an imbalance in resources, as the loss of the scientific workforce further strains the available expertise and infrastructure, hindering the advancement of neuroscience research initiatives. Consequently, the absence of skilled researchers and the knowledge they possess impede the development of a thriving research environment and the ability to address complex neurological challenges. Furthermore, the brain drain perpetuates a global knowledge production and distribution imbalance. Valuable insights and discoveries that could have been generated within Africa are often realised elsewhere, resulting in a potential loss of indigenous perspectives and understanding of neurological disorders specific to the African context. This absence limits the development of tailored interventions, diagnostics, and treatment strategies for prevalent neurological conditions on the continent. The brain drain impacts individual researchers and has broader societal implications. It disrupts collaborative networks, institutional growth, and scientific mentorship opportunities, weakening the foundation for interdisciplinary collaboration and stifling the generation of innovative ideas and approaches to tackle pressing neurological challenges in Africa.

Conducting neuroscience research in diverse cultural contexts like Africa presents significant challenges that necessitate careful attention to sociocultural and ethical considerations. The interplay between cultural norms, informed consent, and ethical concerns within such research involving human participants requires diligent navigation to uphold the integrity and validity of study outcomes. Respecting cultural norms and sensitivities is paramount when researching diverse cultural contexts. Adhering to cultural values, beliefs, and practices fosters trust, facilitates participant engagement, and ensures the inclusion of diverse populations. Researchers must meticulously design studies that align with cultural norms, promoting cultural appropriateness and respect throughout the research process.

Securing informed consent stands as a key ethical consideration. Obtaining informed consent can be complex in diverse cultural contexts due to language barriers, health literacy discrepancies, and varying cultural interpretations of research^16^. Researchers must implement culturally sensitive strategies to facilitate clear communication and comprehension, allowing participants to make autonomous choices. Ethical concerns arise when researching vulnerable populations, including individuals with neurological disorders, children, and marginalised communities. Safeguarding the rights and welfare of these individuals necessitates additional ethical deliberation. Respecting privacy, ensuring confidentiality, and minimising harm are essential considerations. When working with these populations, researchers must adhere to ethical principles such as beneficence, justice, and respect for autonomy.

## Neuroscience education and capacity building

In Africa, neuroscience education and capacity-building initiatives are paramount in advancing scientific knowledge, addressing healthcare challenges, and promoting innovation. The recognition of neuroscience’s critical role in comprehending the intricacies of the brain and neurological disorders has spurred efforts to fortify educational programs and bolster research capabilities.

Central to African neuroscience education is establishing specialised training programs at universities and research institutions. These programs aim to give students a comprehensive understanding of fundamental neuroscience principles, research methodologies, and practical skills. By equipping students with a robust foundation in neuroscience, these initiatives contribute to cultivating a skilled neuroscience workforce in Africa. Collaborative partnerships between African institutions and international organisations have supported neuroscience education. These alliances facilitate knowledge exchange, curriculum development, and access to resources and expertise. Moreover, they provide African students and researchers with opportunities to engage in training programs, workshops, and conferences, both locally and internationally, fostering a broader perspective and facilitating networking opportunities.

Efforts to enhance research capabilities and infrastructure form a crucial aspect of capacity building in neuroscience. These endeavours encompass the provision of state-of-the-art laboratories, advanced neuroimaging equipment, and access to cutting-edge technologies. By investing in research infrastructure, African institutions can foster high-quality research, attract talented researchers, and stimulate scientific advancements within the field of neuroscience. Beyond formal education and infrastructure development, capacity-building initiatives in neuroscience encompass mentorship programs, research grants, and scientific collaborations. Mentorship programs establish connections between seasoned researchers and early-career scientists, offering guidance, support, and avenues for skill development. Research grants from local and international funding agencies enable scientists to pursue innovative research projects and address pressing neurological health issues specific to Africa. Collaborations with international partners facilitate knowledge sharing, interdisciplinary approaches, and the exchange of best practices.

Neuroscience education and capacity building in Africa are pivotal in alleviating the burden of neurological disorders, improving healthcare outcomes, and driving global scientific advancements. By nurturing a skilled workforce, fostering research collaborations, and investing in infrastructure, Africa can leverage its unique strengths to make substantial contributions to neuroscience. Through unwavering commitment and support, Africa has the potential to emerge as a prominent hub for neuroscience research, education, and innovation, revolutionising the understanding and management of neurological conditions.

## Collaborative networks and international partnership

Collaborative networking and international partnerships are crucial in advancing education and research in Africa. The continent boasts several high-quality neuroscience education and research institutions in Egypt, Ghana, South Africa, Nigeria, Tunisia, and Morocco^17^. However, the overall research outputs from these institutions could be improved by more funding and resources for education and research in the region. African researchers often seek collaborations with researchers from other parts of the world to overcome these challenges, particularly in North America, Europe, and Asia. These collaborations provide access to cutting-edge technologies, robust infrastructure, and ample funding, essential for conducting high-quality research and development.

Moreover, researchers from these regions possess a diligent research workforce and have established a strong track record in publishing their work in journals with high-impact factors. By collaborating with international partners, African researchers can improve the quality of their research and enhance its reach, thereby increasing the likelihood of citations and broader recognition. In addition, collaboration with researchers outside Africa proves particularly beneficial for large-scale studies such as systematic reviews and meta-analyses. These studies require access to a significant volume of research articles, which African researchers may have limited access to since many African institutions still need subscriptions to these journals. Collaborating with international partners helps bridge this gap and enables African researchers to access the necessary literature for comprehensive studies.

Several international organisations have been prominent in advancing African education and research. Notable examples include the Teaching and Research in Natural Sciences for Development in Africa (TReND), the International Brain Research Organization (IBRO), and the African Mental Health Research Initiative (AMARI). These organisations provide support, training, and resources to African researchers, fostering capacity building and knowledge exchange in neuroscience. TReND, a nonprofit organisation, plays a crucial role in Africa by supporting the establishment of research institutions, providing funding for diverse neuroscience projects, and organising specialised neuroscience courses for researchers across the continent. Moreover, TReND in Africa offers training programs focused on next-generation sequencing data analysis, equipping scientists with the necessary skills to interpret neurogenomics data. IBRO, as an international entity, has made significant contributions to neuroscience training in Africa by establishing various centres. With the sponsorship of over 50 neuroscience schools and courses, IBRO has trained more than 1000 neuroscientists, fostering capacity building and knowledge exchange^18^. Additionally, they provide essential training in neuroscience, complemented by high-quality equipment and resources, further enhancing the region’s capacity in the field. The African Mental Health Research Initiative (AMARI) is a collaborative organisation of four institutions in Ethiopia, Malawi, South Africa, and Zimbabwe. Through their leadership in high-quality research projects, AMARI has made significant strides in advancing mental healthcare within their respective countries.

Influential organisations in Africa also include the Human Heredity and Health in Africa Initiative (H3 Initiative), which focuses on promoting neuroscience-based genetic studies; the Society of Neuroscientists of Africa (SONA), dedicated to improving neuroscience education; and the Korle Bu Neuroscience Foundation (KBNF), actively engaged in building neuroscience infrastructure in poor African nations. These organisations collectively contribute to advancing neuroscience education, research, and infrastructure in Africa, fostering interdisciplinary collaboration, promoting scientific excellence, and addressing critical healthcare challenges on the continent.

## Recommendations and future directions

Neuroscience research in Africa has shown notable progress in recent years, yet important recommendations and future directions remain to advance the field further. These recommendations include funding, collaborations, capacity building, research infrastructure, sociocultural considerations, translational research, data sharing and open access, and public engagement.

Increasing funding for neuroscience research necessitates the concerted efforts of governments, funding agencies, and philanthropic organisations. A multifaceted approach can be employed to bolster financial support and foster the growth of neuroscience in Africa. Firstly, advocating for increased funding through targeted awareness campaigns and proactive engagement with policymakers is paramount. By illuminating the societal and economic benefits stemming from advancements in neuroscience, stakeholders can raise the profile of the field and garner support. Employing persuasive communication techniques, such as highlighting the potential impact on public health and the potential for scientific innovation, can effectively galvanise support for enhanced financial allocations. In addition, fostering collaborations and forging strategic partnerships among government bodies, funding agencies, and private organisations is crucial. Through such collaborative endeavours, resources can be pooled, expertise can be shared, and the financial burden can be mitigated. This cooperative approach enables a more comprehensive and coordinated effort in securing funding for neuroscience research. Public-private partnerships and joint funding initiatives, facilitated by clear frameworks and efficient communication channels, can effectively augment the financial resources available for neuroscience projects. Another pivotal strategy involves establishing dedicated grants and funding opportunities tailored specifically to neuroscience research in Africa. By delineating clear guidelines, streamlining application processes, and ensuring equitable distribution, governments and funding agencies can promote accessibility and transparency. Such targeted funding mechanisms should encompass various stages of research, including basic science, translational research, and clinical trials. This comprehensive approach ensures that researchers at different stages of their careers and projects of diverse scopes can benefit from the available funding. Similarly, philanthropic support is vital in bolstering funding for neuroscience research. Engaging with philanthropic organisations and individuals demonstrating a vested interest in advancing neuroscience can yield substantial financial contributions. Researchers can effectively engage with philanthropic entities by articulating the value of neuroscience research and its potential societal impact. Cultivating meaningful relationships with these stakeholders can result in substantial funding and long-term partnerships.

Moreover, the significance of collaborations and partnerships cannot be overstated in propelling the advancement of neuroscience research. By fostering synergistic collaborations between African institutions and international research organisations, an exchange of knowledge, technology, and access to advanced research tools can be facilitated. These collaborative endeavours enable African researchers to benefit from international expertise and resources, augmenting the quality and impact of neuroscience research conducted within the continent. Joint research projects, training programs, and workshops are conduits for sharing best practices, refining methodologies, and cultivating a vibrant research ecosystem.

Encouraging collaborative research projects between African institutions and international partners not only expands the scope of the investigation but also promotes the exploration of diverse perspectives and methodologies. This interdisciplinary approach nurtures innovation and fosters a holistic understanding of neuroscience research. By establishing platforms for knowledge exchange and collaboration, such as joint research initiatives and scientific networks, African researchers can actively contribute to and benefit from the global neuroscience community. In parallel, attention must be directed towards capacity building to ensure the growth and sustainability of African neuroscience research. Developing and expanding neuroscience training programs at various educational levels is pivotal in nurturing a skilled and diverse neuroscience workforce. Workshops, conferences, and research training opportunities are invaluable platforms for researchers to enhance their technical expertise, scientific acumen, and critical thinking abilities. By equipping researchers with the requisite skills and knowledge, capacity-building initiatives empower them to contribute effectively to the field and drive meaningful advancements. Furthermore, investing in establishing specialised neuroscience research centres and infrastructure supports expanding research capabilities within Africa. These centres can serve as hubs for collaborative research, providing researchers with state-of-the-art facilities, advanced equipment, and cutting-edge technologies. By bolstering infrastructure, African institutions can attract and retain talented researchers, create a vibrant scientific environment, and catalyse groundbreaking discoveries in neuroscience.

Improving research infrastructure is vital for conducting high-quality neuroscience research. Investment in state-of-the-art laboratories, specialised equipment, and advanced technologies enable researchers to perform sophisticated experiments and analyses. Collaborating with institutions with advanced research infrastructure can help bridge resource gaps and elevate the quality of African research. In addition, sociocultural considerations are essential in conducting neuroscience research within diverse African contexts. Active engagement with local communities, respect for cultural norms, and adherence to ethical practices are critical when conducting research involving human participants. Collaborating with local stakeholders can help address sociocultural challenges and ensure the societal relevance of neuroscience research.

Translational research should be prioritised, focusing on the practical application of scientific discoveries in healthcare. By addressing prevalent neurological conditions and healthcare challenges African populations face, researchers can develop diagnostics, treatments, and interventions that are contextually relevant, accessible, and culturally appropriate. Data sharing and open access are vital for collaboration, reproducibility, and knowledge dissemination. Establishing data repositories and platforms that facilitate the sharing of neuroscience data within the African research community can foster scientific progress. Advocating for policies that support open-access publishing can maximise the impact of neuroscience research in Africa.

Public engagement is pivotal in engendering heightened awareness and comprehensive comprehension of neuroscience research. Science communication initiatives play a vital role in disseminating knowledge and enlightening the public regarding the profound significance of neuroscience and its potential implications for healthcare. In Africa, fostering a culture of public engagement becomes instrumental in advocating for increased research funding, influencing policy decisions, and seamlessly integrating neuroscience into healthcare practices. To accomplish this, establishing a robust platform for dialogue among researchers, policymakers, and the public assumes paramount importance.

Effective science communication initiatives are instrumental in enlightening and educating the public about the intricacies and implications of neuroscience research. By employing accessible language and captivating mediums, such initiatives can effectively communicate complex scientific concepts to non-experts. Utilising visual aids, interactive demonstrations, and engaging public lectures, science communicators can bridge the gap between the scientific community and the general public, elucidating the significance of neuroscience research and its potential impact on healthcare outcomes.

In Africa, advocating for increased research funding through public engagement is imperative. Researchers and science communicators can garner public support and galvanise momentum for adequate funding by fostering a sense of shared responsibility and emphasising the societal benefits. Engaging with local communities, organising public forums, and leveraging social media platforms can be employed to actively involve the public in neuroscience research, enabling them to understand the profound importance of funding for scientific advancements in this field. Furthermore, integrating neuroscience into policy decisions and healthcare practices necessitates an inclusive approach that embraces dialogue among researchers, policymakers, and the public. Collaborative platforms, such as stakeholder meetings, policy roundtables, and public consultations, facilitate knowledge exchange, consensus building, and the development of evidence-based policies. By involving policymakers and the public in these discussions, Africa can ensure that the perspectives and insights of all stakeholders are considered, leading to more informed and effective policy decisions that prioritise neuroscience research and its implications for healthcare.

## Conclusion

Neuroscience research in Africa holds immense potential for advancing our understanding of the brain and addressing the healthcare challenges the continent faces. Throughout this paper, we have examined the significant progress made in recent years, despite the challenges of limited funding, inadequate infrastructure, brain drain, and sociocultural considerations. This progress has been possible due to the concerted efforts of researchers, institutions, and international collaborations.

Embracing recommendations that shape the future of neuroscience research in Africa is imperative. Increasing funding and resources, fostering collaborations and partnerships, investing in capacity building and research infrastructure, considering sociocultural factors, promoting translational research, advocating for data sharing and open access, and engaging the public are crucial. Implementing these recommendations can position Africa as a global leader in neuroscience research, contributing to the scientific understanding of the brain and improving healthcare outcomes for its populations.

The future of neuroscience research in Africa shines brightly. With continued support, it has the potential to make significant contributions to global scientific knowledge and transform neurological healthcare on the continent and beyond. By championing the growth of neuroscience in Africa, the necessary funding, resources, and collaborations can be put in place to propel research forward. Africa can shape a future where breakthrough discoveries and advancements in neuroscience benefit the well-being of individuals and communities across Africa and the world.

## Ethical approval

Not applicable.

## Consent

Not applicable.

## Sources of funding

No external funding was received for this study.

## Author contribution

G.O. and N.A.: Conceptualization. All authors: writing.

## Conflicts of interest disclosure

The authors declare that they have no conflicts of interest.

## Research registration unique identifying number (UIN)

Not applicable.

## Guarantor

Nicholas Aderinto.

## Provenance and peer review

Not commissioned, externally peer-reviewed.

## Data availability statement

Data sharing is not applicable to this perspective article.
